# Of cheese and bedsheets – some notes on correlation

**DOI:** 10.3325/cmj.2020.61.293

**Published:** 2020-06

**Authors:** Pero Hrabač, Vladimir Trkulja

**Affiliations:** 1 Department of Medical Statistics, Epidemiology, and Medical Informatics, “Andrija Štampar” School of Public Health, University of Zagreb School of Medicine, Zagreb, Croatia *phrabac@hiim.hr*; 2 Department of Pharmacology, Zagreb University School of Medicine, Zagreb, Croatia

A somewhat bizarre title of this column comes from one of the charts shown on the website named “Spurious Correlations” (1). It shows the correlation or association between two variables. The variables in question are *per capita* cheese consumption and the number of people who died by becoming entangled in their bedsheets. The graph clearly shows that the increase in cheese consumption is closely followed by the increase in the number of people who died in the previously described way. It is clear that there cannot be any causal link between the variables, ie, the change of one variable does not cause the change of the other variable. However, it is also indisputable that there is a correlation between the two variables and that such correlation can be expressed numerically, regardless of whether it makes sense or not. This type of correlation is called linear, bivariate, or Pearson correlation, after one of the founders of modern statistical science, Karl Pearson.

K. Pearson is also known as C. Pearson (his real name was Carl, he later changed it to Karl), or as “Pearson father” because his son, Egon Pearson, was also a statistician. Moreover, E. Pearson succeeded his father as head of the Applied Statistics Department at University College London. Born in 1857 into a well-to-do London family (his father was a well-known lawyer and his mother a ship-owner’s daughter), K. Pearson was a man of very wide interests. Although he graduated in mathematics at King's College, Cambridge, at the age of 22, his early professional interest was social Darwinism, and he was also a prominent promoter of eugenics. Thanks to a success in his studies, he received a 6-year college fellowship, which enabled him to continue his education in Heidelberg and Berlin (2), where he studied physics and philosophy. Describing the breadth of his interests, he said: “*I rush from science to philosophy, and from philosophy to our old friends the poets; and then, over-wearied by too much idealism, I fancy I become practical in returning to science*.“ (3) After returning to London, he first studied law for a while and then returned to his original profession, working as a professor of mathematics and applied mathematics and mechanics at University College in London. It was only relatively late in his professional career (in 1893, when he was 36) that he began to take a more active interest in statistics. Namely, in 1891 he accepted the position of professor of geometry at Gresham College in London where he met the zoologist W.F.R. Weldon. Weldon was an evolutionary biologist with a keen interest in mathematical and statistical theory (4). Among other things, Weldon is known for his famous experiment in which he rolled a set of 12 dice for 23,306 times and recorded each of the results (5). K. Pearson later used these data in his work on the chi-square test (6). Weldon had a crucial impact on changing Pearson’s area of interest and bringing his focus to statistics. The problem Weldon encountered was the application of statistical and mathematical methods developed by F. Galton in the field of heredity and eugenics. Weldon therefore introduced Pearson to Galton, who was a cousin of C. Darwin (they had a common grandparent, Erasmus Darwin). Galton became Pearson’s mentor and friend until his death in 1911. Pearson continued Galton’s work as the Galton Chair of Eugenics (later the Galton Chair of Genetics), a chair that still exists today, as part of the Department of Biology at University College London (7). For the next fifteen years, working on the problems of evolutionary biology and eugenics, he laid the foundations of modern statistical science. One of the first areas in statistics that developed strongly as a result of his work was the theory of correlation, which today bears his name and is commonly known as Pearson's correlation. Unlike Galton, who developed basic concepts of correlation and regression in heredity, but was primarily interested in physiological and hereditary mechanisms, Pearson approached the problem of association mathematically, uninterested in the biological aspects. The subject of his interest was how to find a mathematical connection between observed values, regardless of what mechanisms lead to such a connection (2).

What was the problem Pearson was trying to solve and what did the solution look like? The answer to these questions is also the definition of the assumptions the data must meet to be suitable for a Pearson correlation analysis.

First, the data must be in pairs, with each x having a value of y belonging to it (and only to it). For example, in a pregnancy study, one might find that a gestational age of 37 weeks (x) was associated with a birth weight of 3650 g (y), a gestational age of 34 weeks with a weight of 3480 g, and an age of 41 weeks with a weight of 4030 g. From the given example, the second assumption is notable as well – the variables must be quantitative, measured on an interval or ratio scale. Pearson correlation coefficient then represents the measure of strength and direction of correlation. The direction is in this case positive because a longer duration of gestation is associated with a higher birth weight. If we were to look at a different example, eg, the number of cigarettes smoked per day and life expectancy, we would probably find a negative association. This is because more cigarettes smoked will most likely be associated with a shorter life expectancy on average. Both parameters, ie, the strength of the connection as well as its direction, are represented in a parameter known as “Pearson's r”. Pearson r can take values from -1 to 1 and is calculated by a very simple formula:


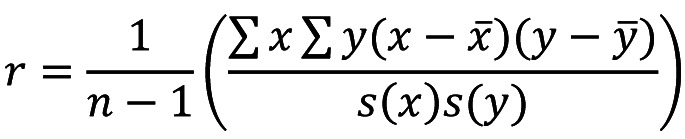


In the above formula we include pairs of observed values as follows:

n = number of observations (number of measurements, number of pairs)


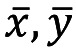
 = means for x and y

s(x), s(y) = standard deviations for x and y.

Consider a simple example. Three students were preparing for a written exam. Pero studied for 3 days and scored 76 points. Vlado studied for 8 days and scored 77 points, while Svjetlana studied for 14 days and scored 97 points.

In the above formula we substitute:

n = 3 (there are three pairs of observations)

The denominator of the fraction is obtained by calculating the difference from the mean value for each x and y, multiplying and summing the results. The means for x and y are as follows:


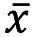
= (3 + 8 + 14)/3 = 8.33


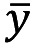
 = (76 + 77 + 97)/3 = 83.33

The fraction numerator is then:

[(3 – 8.33) × (76 – 83.33)] + [(8 – 8.33) × (77 – 83.33)] + [(14 – 8.33) × (97 – 83.33)] =

 = [(-5.33) × (-7.33)] + [(-0.33) × (-6.33)] + [(5.67) × (13.67)] =

 = 39.07 + 2.09 + 77.51 =

 = 118.67

The denominator is calculated as the product of standard deviations. With standard deviations being:

s(x) = 5.51

s(y) = 11.84

the denominator of the fraction is 5.51 × 11.84 = 65.24

The formula then looks like:


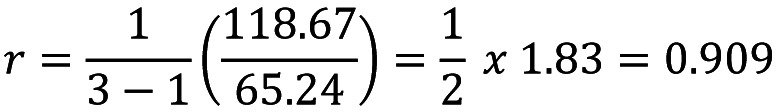


We can conclude that the correlation coefficient (r) equals 0.909. The following should also be noted: r and r squared (see below) would be identical had we defined “test result” as X and “days spent studying” as Y – that is, the result of the correlation analysis is the same regardless of which variable is defined as X and which is defined as Y, reflecting the previous point that Pearson was focused simply on finding a mathematical solution to quantify the strength of association. The same goes for the example of birthweight and gestational age.

Since the value of r represents effect size, it can be interpreted based on certain limits, for example (8):

0.00-0.19: “very weak” association between x and y (and vice-versa)

0.20-0.39: “weak,”

0.40-0.59: “moderate,”

0.60-0.79: “strong,”

0.80-1.0: “very strong.”

One should also keep in mind:

• The correlation coefficient r defines the strength of the association. The squared value of r (r^2^ or the coefficient of determination; in the above case 0.909 × 0.909 = 0.826) represents the proportion of the variance of one variable that can be explained by another variable.

• High r value still says nothing about the causality of the correlation between the variables. The fact that values correlate does not mean that a change in one variable causes a change in another. It is always possible that there is a variable that we do not measure, or the one we are not even aware of, and that this variable is the real cause of the correlation.

• If the relationship between the variables is not linear (ie, if it cannot be adequately approximated by a straight line), analysis of correlation will lead to erroneous conclusions.

• If there are outliers, ie, values that differ greatly in one direction or another, this will significantly affect the results. For example, if the above example included a fourth student who studied for only one day but received 100 points on the test, the value of r would decrease from 0.909 to 0.121. The same is true if we include data for a student who studied for a long time and received only a few points. We say that Pearson correlation is very sensitive to outliers or, more generally, that the results will not be reliable if the values of both variables do not follow the normal distribution.

For these reasons, and others that go beyond the scope of this column, in the following issue we will consider in more detail the relationship between correlation and regression with special reference to the causality of relationships between variables and multivariate methods of analyzing larger data sets.
